# Identified single-nucleotide polymorphisms and haplotypes at 16q22.1 increase diabetic nephropathy risk in Han Chinese population

**DOI:** 10.1186/s12863-014-0113-8

**Published:** 2014-10-31

**Authors:** Li-Na Liao, Ching-Chu Chen, Fang-Yang Wu, Cheng-Chieh Lin, Jen-Hao Hsiao, Chwen-Tzuei Chang, Sharon LR Kardia, Tsai-Chung Li, Fuu-Jen Tsai

**Affiliations:** Department of Public Health, College of Public Health, China Medical University, Taichung, Taiwan; Division of Endocrinology and Metabolism, Department of Medicine, China Medical University Hospital, Taichung, Taiwan; School of Chinese Medicine, College of Chinese Medicine, China Medical University, Taichung, Taiwan; Department of Family Medicine, China Medical University Hospital, Taichung, Taiwan; School of Medicine, College of Medicine, China Medical University, Taichung, Taiwan; Bioinformatics and Biostatistics Core, Center of Genomic Medicine, National Taiwan University, Taipei, Taiwan; Department of Epidemiology, University of Michigan, Ann Arbor, MI USA; Graduate Institute of Biostatistics, College of Management, China Medical University, No. 91 Hsueh-Shih Road, Taichung, 40402 Taiwan; Department of Healthcare Administration, College of Health Science, Asia University, Taichung, Taiwan; Department of Medical Genetics, China Medical University Hospital, Taichung, Taiwan; Department of Biotechnology and Bioinformatics, Asia University, Taichung, Taiwan

**Keywords:** Diabetic nephropathy, Single-nucleotide polymorphism, Haplotype, Han Chinese

## Abstract

**Background:**

Diabetic nephropathy (DN) has become one of the most common causes of end-stage renal disease (ESRD) in many countries, such as 44.5% in Taiwan. Previous studies have shown that there is a genetic component to ESRD. Studies attempting to determine which genetic variants are related to DN in Han Chinese are limited. A case–control study was conducted to identify DN susceptibility variants in Han Chinese patients with type 2 diabetes.

**Results:**

We included 574 unrelated type 2 diabetes patients (217 DN cases and 357 controls), who were genotyped using Illumina HumanHap550-Duo BeadChip. In single-SNP association tests, the SNPs rs11647932, rs11645214, and rs6499323 located at 16q22.1 under the additive-effect disease model were significantly associated with an approximately 2-fold increased risk of DN. In haplotype association tests, identified haplotypes located in the chromosome 16q22.1 region (containing *ST3GAL2*, *COG4*, *SF3B3*, and *IL34* genes) raised DN risk. The strongest association was found with haplotype rs2288491-rs4985534-rs11645214 (C-C-G) (adjusted odds ratio [AOR] 1.93, 95% confidence interval [CI] 1.83-2.03, p = 6.25 × 10^−7^), followed by haplotype rs8052125-rs2288491-rs4985534-rs11645214 (G-C-C-G) (AOR 1.92, 95% CI 1.82-2.02, p = 6.56 × 10^−7^), and haplotype rs2303792-rs8052125-rs2288491-rs4985534-rs11645214 (A-G-C-C-G) (AOR 1.91, 95% CI 1.81-2.01, p = 1.15 × 10^−6^).

**Conclusions:**

Our results demonstrate that the novel SNPs and haplotypes located at the 16q22.1 region may involve in the biological pathways of DN in Han Chinese patients with type 2 diabetes. This study can provide new insights into the etiology of DN.

**Electronic supplementary material:**

The online version of this article (doi:10.1186/s12863-014-0113-8) contains supplementary material, which is available to authorized users.

## Background

Diabetic nephropathy (DN) has become one of the most common causes of end-stage renal disease (ESRD) in many countries. In Taiwan, diabetes accounted for 44.5% of all new cases of ESRD [1], and more than 99% diabetes patients were with type 2 diabetes [2]. Diabetic ESRD patients had worse survival than non-diabetic ESRD patients [3].

It has been reported that genetic predisposition is one of the main risk factors for the development of DN [4]. Numerous familial aggregation studies have suggested that genetic susceptibility plays an important role in the development and progression of DN [5,6]. Epidemiologic studies have shown that 35% of patients with diabetes develop nephropathy, irrespective of glycemic control [7,8].

Mooyaart et al. performed a meta-analysis to evaluate the pooled effect of each genetic variant reproducibly associated with DN [9]. They reported that 21 of 34 replicated genetic variants remained significantly associated with DN. These 34 variants were in or near the following genes: *ACE*, *ELMO1*, *PPARG*, etc. Recently, several genome-wide association studies (GWASs) have attempted to detect genetic variants associated with the risk of DN or diabetic ESRD in those of Japanese [10], Pima Indian [11], and African American [12] with type 2 diabetes, as well as European ancestry [13,14] with type 1 diabetes.

Studies attempting to determine which genetic variants are related to DN in Han Chinese patients with type 2 diabetes are limited. In the present genetic association study, a case–control study was carried out to identify DN susceptibility variants in Han Chinese patients with type 2 diabetes, which can provide new insights into the etiology of DN.

## Methods

### Study subjects

Individuals with type 2 diabetes and aged over 20 years were recruited using the American Diabetes Association (ICD-9-CM, Diagnosis code 250) criteria for diagnosis of type 2 diabetes. Individuals with type 1 diabetes, gestational diabetes, and maturity-onset diabetes of the young were excluded. The dataset used is part of the whole dataset for the published paper titled “A Genome-wide Association Study Identifies Susceptibility Variants for Type 2 Diabetes in Han Chinese” [15]. A total of 995 type 2 diabetes subjects recruited from China Medical University Hospital were included in the current study. All patients with type 2 diabetes were of Han Chinese origin, including Minnan, Hakka, and Mainland Chinese. Individuals with significant aboriginal ancestry were excluded by using self-administered questionnaires with six items regarding the ancestral origin of their parents and grandparents. Chronic kidney disease (CKD) was determined by estimated glomerular filtration rate (eGFR) and urine protein. Diabetic patients with eGFR <60 mL/min/1.73 m^2^ or with proteinuria determined by a spot urine dipstick of >1+ were defined as DN cases. A total of 217 DN cases were eligible for the study. To increase comparability between DN cases and controls, 357 controls were randomly selected based on frequency-matching of their age and durations of diabetes. This study was approved by the Human Research Committee of China Medical University Hospital. All patients signed informed consent forms.

### Measurements

Blood samples were collected in the morning after a 12-h overnight fast and were sent for analysis within 4-h of collection. Spot morning urine samples were collected. Triglycerides, total cholesterol, high-density lipoprotein cholesterol (HDL-C), low-density lipoprotein cholesterol (LDL-C), creatinine, blood urea nitrogen (BUN), and uric acid were measured by a biochemical autoanalyser (Beckman Coulter, Synchron LX20, Fullerton, CA, USA). Renal function was evaluated by eGFR, which was estimated by using the Modification of Diet in Renal Disease Study equation for Taiwanese: eGFR (ml/min/1.73 m^2^) = 175 × (serum creatinine (mg/dL)^−1.154^ × (age)^−0.203^ × (0.742 if female) × 0.945 [16]. We also used the Chronic Kidney Disease Epidemiology Collaboration equation to obtain eGFR [17]. The proportions of eGFR <60 mL/min/1.73 m^2^ by using above two equations were similar. A spot urine dipstick test was used for detecting proteinuria. Patients with a positive dipstick test (>1+) were classified as with proteinuria [18]. The sociodemographic and lifestyle characteristics and the self-reported health status for each subject were recorded using self-administered questionnaires.

### Genotyping and quality control

Genomic DNA from peripheral blood was prepared using the Puregene DNA isolation kit (Gentra Systems, Minneapolis, MN, USA). Our samples were genotyped using Illumina HumanHap550-Duo BeadChip, which was performed by deCODE Genetics (Reykjavík, Iceland). Genotypes were called using the standard procedure in BeadStudio (Illumina, Inc., San Diego, CA, USA), with the default parameters recommended by the platform manufacturer. The genotyping quality control procedures used to identify and remove poor-quality data were described previously [15]. Individual SNPs were excluded if they had a total call rate <95% across all individuals, a minor allele frequency <5% and a total call rate <99%, or had significant deviation from Hardy-Weinberg disequilibrium (p-value <10^−7^) in these subjects. Further details of genotyping quality control procedures are available in the published study [15]. After SNPs quality control, 429,018 SNPs were used and their total call rate was 99.9%. We also examined population stratification by using multidimensional scaling (MDS) analysis as implemented in PLINK. The results of MDS analysis showed that there was no evidence for population stratification.

### Statistical analysis

Demographic and clinical characteristics of study subjects were examined, including sex, age, diabetes-related variables, behaviors, biochemical variables, and history of diseases. Continuous variables are reported as mean ± standard deviation (SD), and categorical variables are reported as number and percentage. Two-sample t tests and Chi-square tests were used for the bivariate analyses. Because the distribution of the triglycerides was skewed, the data were normalized using a natural log-transformation, and the geometric mean ± SD was calculated. To identify the DN susceptibility variants, single-SNP association tests using Cochran-Armitage trend test were performed. Then, multiple logistic regression analysis using an additive-effect disease model (an ordinal genotype model: 0, 1 and 2 of a minor allele) was performed for each SNP with adjustment of the subject's sex, age, BMI, and durations of diabetes. The Bonferroni correction was used to adjust for multiple comparisons. P-value less than 10^−4^, association was considered to be statistically significant. According to previously published studies, as well as the results of single-SNP association tests and the Manhattan plot in our current study, potential susceptibility regions for DN were selected to perform haplotype analysis. In haplotype-based association analysis, the sliding window approach was adopted to detect haplotype effects. The window sizes of 3-SNP, 4-SNP, and 5-SNP haplotypes were used. Each haplotype with a frequency of >0.05 in this population was analyzed. Odds ratios (ORs) and their corresponding 95% confidence intervals (CIs) were calculated to estimate the effect sizes of the identified SNPs and haplotypes. In addition, the linkage disequilibrium (LD) structures of the identified contiguous SNPs were examined. Pairwise LD was measured by the *r*^2^ statistic. For power calculation of our case–control study (217 DN cases and 357 controls), Quanto software [19] was used. Under an additive effect disease model with a prevalence of 10% for DN (from our dataset), given a genetic relative risk of 1.85 and a disease allele frequency of 0.25-0.45, the power of our study was 0.76-0.86 at an alpha level of 10^−4^. All analyses were carried out using Haploview (v4.2) [20], PLINK (v1.07) (pngu.mgh.harvard.edu/purcell/plink) [21], and SAS (v9.3, SAS Institute Inc, Cary, NC, USA) software. The regional plot was plotted from the LocusZoom, a web-based plotting tool (csg.sph.umich.edu/locuszoom) [22]. The *in silico* prediction tool is-rSNP was used to predict potential regulatory SNPs (rSNPs) [23].

## Results

### Demographic and clinical characteristics of study samples

A total of 574 type 2 diabetes study participants, comprising 217 cases of DN and 357 type 2 diabetes patients without DN controls, were included in the analysis. Table 1 shows the sociodemographic factors, diabetes-related variables, lifestyle behaviors, biochemical variables, and history of diseases according to DN status. The mean age of the participants was about 62 years for both groups and their mean HbA1c at enrollment was 8.2 ± 1.7% for the cases and 7.9 ± 1.4% for the controls. The mean durations of diabetes were 11.1 ± 7.7 years for the cases and 10.2 ± 7.2 years for the controls. Compared with the controls, DN cases had higher mean BMI, triglycerides, creatinine, uric acid, BUN, and prevalence of hypertension and heart disease.

**Table 1:** **Demographic and clinical characteristics of study samples**

**Characteristics**	**Cases (n = 217)**	**Controls (n = 357)**	**p-value**
**Sociodemographic factors**			
Male	108 (49.8)	170 (47.6)	0.617
Age at enrollment (years)	62.9 ± 9.1	62.6 ± 9.2	0.673
**Diabetes-related variables**			
Age at onset of type 2 diabetes (years)	51.8 ± 9.4	52.4 ± 9.1	0.463
Durations of diabetes (years)	11.1 ± 7.7	10.2 ± 7.2	0.154
HbA1c at enrollment (%)	8.2 ± 1.7	7.9 ± 1.4	0.043
BMI (kg/m^2^)	26.0 ± 3.9	24.7 ± 3.5	< 0.001
**Lifestyle behaviors**			
Regular exercise	123 (56.7)	260 (72.8)	< 0.001
Smoking status			0.926
Ever	33 (15.2)	52 (14.6)	
Yes	36 (16.6)	56 (15.7)	
Alcohol drinking			0.032
Ever	20 (9.2)	14 (3.9)	
Yes	34 (15.7)	63 (17.7)	
**Biochemical variables**			
Triglycerides (mg/dL)^a^	156.8 ± 1.8	131.1 ± 1.8	< 0.001
Total cholesterol (mg/dL)	192.8 ± 53.8	185.9 ± 37.2	0.099
HDL-C (mg/dL)	47.0 ± 13.9	49.4 ± 13.7	0.042
LDL-C (mg/dL)	118.1 ± 42.2	117.5 ± 33.3	0.856
Creatinine (mg/dL)	1.4 ± 1.1	0.7 ± 0.2	< 0.001
Uric Acid (mg/dL)	7.4 ± 1.9	5.9 ± 1.4	< 0.001
BUN (mg/dL)	24.9 ± 12.7	15.4 ± 4.0	< 0.001
Proteinuria	125 (57.9)	0 (0.0)	< 0.001
eGFR <60 ml/min/1.73 m^2^	149 (68.7)	0 (0.0)	< 0.001
**History of diseases**			
Hypertension	140 (64.5)	156 (43.7)	< 0.001
Heart disease	67 (30.9)	64 (17.9)	< 0.001
Stroke	17 (7.8)	15 (4.2)	0.066
Diabetic retinopathy	41 (18.9)	41 (11.5)	0.014
Diabetic foot	9 (4.2)	5 (1.4)	0.039

Data were presented as mean ± SD for continuous variables or n (%) for categorical variables. eGFR: estimated glomerular filtration rate; BUN: blood urea nitrogen.

^a^Geometric mean was presented.

### The graphical summary plot and single-SNP association tests

**Figure 1:**
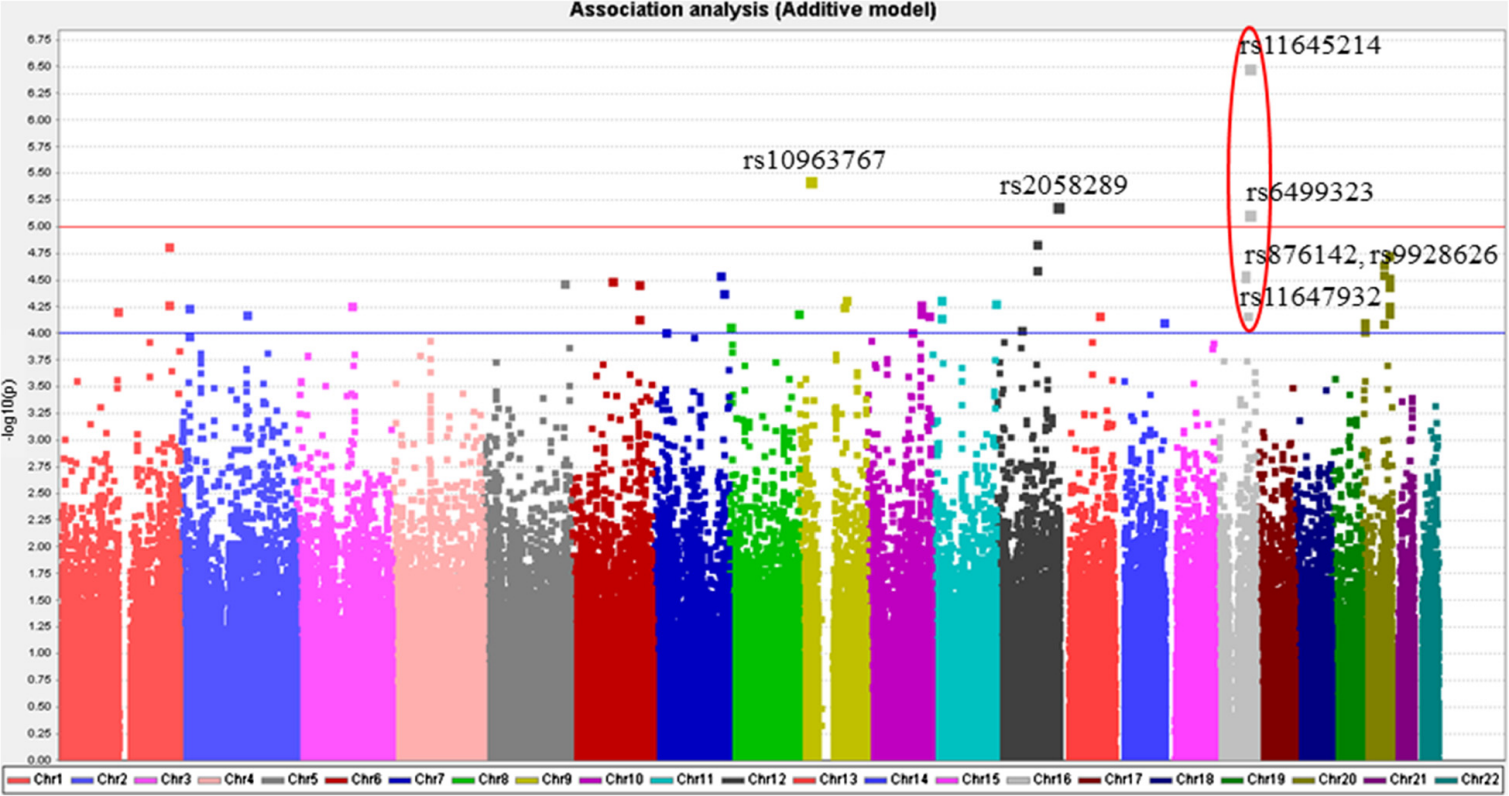
**Graphical summary of DN in single-SNP association analysis under additive-effect disease model.**

The graphical summary plot of DN in single-SNP association analysis under additive-effect disease model is shown in Figure 1. There were four SNPs with p-value <10^−5^ (above the red line), including rs10963767 (intron; *ADAMTSL1* gene; 9p21.3), rs2058289 (intergenic; 12q24.3), rs11645214 (3’ UTR; *SF3B3* gene; 16q22.1), and rs6499323 (intron; *IL34*; 16q22.1). Furthermore, two signals with p-value <10^−4^ were observed, indicating the SNPs around this areas may be potential susceptibility variants for DN; one signal (rs876142, rs9928626, rs11647932, rs11645214, and rs6499323) was on chromosome 16 (16q21-16q22.1 regions) and the other (rs1028555, rs4815800, rs6065925, rs6131015, rs4812997, rs6074024, rs6127983, rs182784, rs1885580, rs7273764, rs6127999, rs6014975, rs4811839, rs6025517, and rs2426712) was on chromosome 20 (20p12.3 and 20q13.1-20q13.3 regions). Among these 15 SNPs on chromosome 20, only SNPs rs6127983 and rs182784 (intron; *BMP7* gene; 20q13.3) and SNPs rs4811839 and rs6025517 (intron; *RAE1* gene; 20q13.3) were inside of genes. A previous study reviewed in a meta-analysis by Mooyaart et al. showed that the Hp 1/2 variant in the *HP* gene (located at 16q22.2) was associated with DN. Therefore, a total of 383 genotyped SNPs at the 16q22.1-16q22.2 regions were selected for this study. Significant SNPs (p-value <10^−4^) identified from single-SNP association analysis, along with the other 8 SNPs identified from haplotype association analysis, are presented in Table 2. The SNP with the lowest p-value (6.25 × 10^−7^) was rs11645214 located on chromosome 16q22.1. This SNP, with a higher risk of DN (adjusted odds ratio [AOR] 1.94, 95% confidence interval [CI] 1.49-2.51), is inside the *SF3B3* gene (Additional file 1: Figure S1). Two other SNPs that showed a significant or borderline significant association with DN, each with an additive-effect, were rs6499323 (*IL34*) and rs11647932 (*ST3GAL2*) on chromosome 16q22.1 (Additional file 1: Figure S1). No significant variants were detected for DN at the 16q22.2 region.

**Table 2:** **Single-SNP association analysis for DN at the 16q22.1-16q22.2 regions under the additive model** ^**a**^

**SNP**	**Minor (major) allele**	**MAF (Cases)**	**MAF (Controls)**	**Gene**	**Location**	**Crude OR (95% CI)**	***p*** **-value** ^**b**^	**Adjusted OR (95% CI)**	***p*** **-value** ^**c**^
rs4985526	T (C)	0.26	0.33	ST3GAL2	intron	0.71 (0.55-0.93)	1.26E-02	0.72 (0.55-0.95)	1.79E-02
**rs11647932**	T (C)	0.24	0.14	ST3GAL2	intron	1.84 (1.36-2.50)	6.39E-05	1.82 (1.34-2.48)	1.37E-04
rs8062183	A (G)	0.35	0.45	ST3GAL2	near-gene-5	0.68 (0.53-0.87)	2.19E-03	0.69 (0.53-0.88)	3.55E-03
rs2303792	G (A)	0.35	0.44	COG4	intron	0.68 (0.53-0.88)	2.54E-03	0.69 (0.53-0.89)	4.05E-03
rs8052125	T (G)	0.35	0.44	COG4	intron	0.67 (0.52-0.86)	1.68E-03	0.68 (0.52-0.87)	2.79E-03
rs2288491	T (C)	0.35	0.44	SF3B3	intron	0.68 (0.53-0.88)	2.62E-03	0.69 (0.53-0.89)	3.91E-03
rs4985534	T (C)	0.24	0.31	SF3B3	intron	0.69 (0.53-0.90)	6.91E-03	0.69 (0.52-0.91)	8.23E-03
**rs11645214**	G (A)	0.49	0.33	SF3B3	3' UTR	1.92 (1.49-2.48)	3.07E-07	1.94 (1.49-2.51)	6.25E-07
**rs6499323**	G (A)	0.47	0.34	IL34	intron	1.77 (1.38-2.28)	7.24E-06	1.81 (1.40-2.35)	7.10E-06
rs7197333	C (T)	0.16	0.21	IL34	intron	0.71 (0.52-0.97)	2.86E-02	0.69 (0.51-0.95)	2.20E-02
rs1006985	C (T)	0.44	0.50	IL34	intron	0.76 (0.60-0.97)	2.85E-02	0.75 (0.58-0.96)	2.18E-02

MAF: minor allele frequency.

^a^Significant SNPs (p-value <10^−4^) identified from single-SNP association analysis, along with the other 8 SNPs identified from haplotype association analysis, are presented.

^b^p-value from Chi-square test (Cochran-Armitage trend test).

^c^p-value from logistic regression under an additive model (an ordinal genotype model: 0, 1 and 2 of a minor allele) after adjustment of subject's sex, age, BMI, and durations of diabetes.

### The LD structures and haplotypic association tests

**Figure 2:**
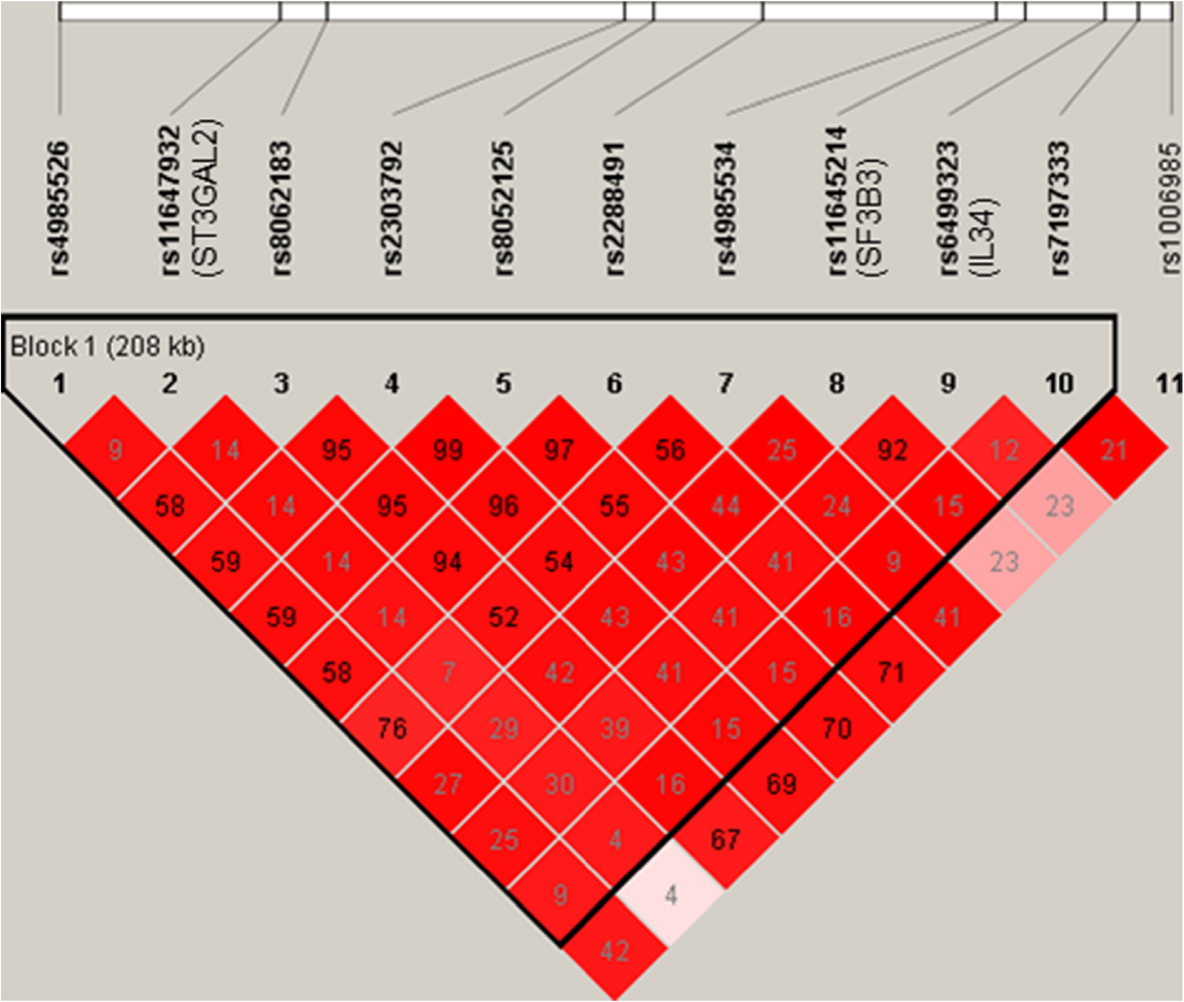
**Pairwise linkage disequilibrium (LD) among the 11 SNPs located at 16q22.1 region.** Pairwise LD was measured by the *r* ^2^ statistic. An *r* ^2^ value of 1 indicates a complete LD between two SNPs, and an *r* ^2^ value of 0 indicates a perfect equilibrium. Lower *r* ^*2*^ value shows a lower degree of LD. Pairwise *D*’ values along with LOD scores were displayed in different colors. White indicates *D*’ <1 and LOD <2; blue indicate *D*’ = 1 and LOD <2; bright red indicate *D*’ = 1 and LOD ≥2; shades of pink/red indicate *D*’ <1 and LOD ≥2.

Pairwise LDs among the 11 SNPs located at 16q22.1 region are shown in Figure 2. SNPs rs8062183 in *ST3GAL2*, rs2303792 and rs8052125 in *COG4*, and rs2288491 in *SF3B3* were in strong LD with each other (*r*^2^ > 0.94). SNP rs11645214 in *SF3B3* was highly in LD with rs6499323 in *IL34* (*r*^2^ = 0.92). Nevertheless, SNP rs11647932 and other SNPs were not in LD with *r*^2^ values ranging from 0.04 to 0.30.

Genotyped SNPs at the 16q22.1-16q22.2 regions were selected for the haplotypic association tests in this study. The window sizes of 3-SNP, 4-SNP, and 5-SNP haplotypes were used to detect haplotype effects on DN, and a frequency of >0.05 was analyzed. The haplotypes with a p-value <10^−4^ are listed in Table 3. There were 4, 3, and 4 haplotypes with p-value <10^−5^ in window size of 3-SNP, 4-SNP, and 5-SNP significantly associated with DN in this sample, respectively, after adjusted for sex, age, BMI, and durations of diabetes. In particular, haplotype rs2288491-rs4985534-rs11645214 (C-C-G) (AOR 1.93, 95% CI 1.83-2.03, p = 6.25 × 10^−7^), haplotype rs8052125-rs2288491-rs4985534-rs11645214 (G-C-C-G) (AOR 1.92, 95% CI 1.82-2.02, p = 6.56 × 10^−7^), and haplotype rs2303792-rs8052125-rs2288491-rs4985534-rs11645214 (A-G-C-C-G) (AOR 1.91, 95% CI 1.81-2.01, p = 1.15 × 10^−6^) seem to be associated with a higher risk of DN. All haplotypes with significant associations with DN consisted of SNPs that exerted significant associations with DN in single-SNP association analysis.

**Table 3:** **Haplotypic association tests for DN at the 16q22.1-16q22.2 regions** ^**a**^

**Haplotypes** ^**b**^	**SNPs**	**H-freq (Cases)**	**H-freq (Controls)**	**Crude OR (95% CI)**	***p*** **-value** ^**c**^	**Adjusted OR (95% CI)**	***p*** **-value** ^**d**^
Window size of 3-SNP							
H1 (C-T-G)	rs4985526-**rs11647932**-rs8062183	0.24	0.14	1.86 (1.72-2.01)	7.36E-05	1.84 (1.70-1.99)	1.10E-04
H2 (T-G-A)	**rs11647932**-rs8062183-rs2303792	0.24	0.14	1.85 (1.71-2.00)	7.18E-05	1.84 (1.70-1.99)	1.07E-04
H3 (C-C-G)	rs2288491-rs4985534-**rs11645214**	0.49	0.33	1.92 (1.82-2.02)	5.40E-07	1.93 (1.83-2.03)	6.25E-07
H4 (C-G-G)	rs4985534-**rs11645214**-**rs6499323**	0.47	0.33	1.86 (1.76-1.96)	2.08E-06	1.90 (1.80-2.01)	1.48E-06
H5 (G-G-T)	**rs11645214**-**rs6499323**-rs7197333	0.47	0.33	1.86 (1.76-1.96)	2.08E-06	1.90 (1.80-2.01)	1.48E-06
H6 (G-T-T)	**rs6499323**-rs7197333-rs1006985	0.39	0.27	1.73 (1.62-1.84)	4.42E-05	1.85 (1.74-1.97)	8.93E-06
Window size of 4-SNP							
H7 (T-G-A-G)	**rs11647932**-rs8062183-rs2303792-rs8052125	0.24	0.14	1.85 (1.71-2.00)	8.22E-05	1.85 (1.71-2.00)	8.04E-05
H8 (G-C-C-G)	rs8052125-rs2288491-rs4985534-**rs11645214**	0.48	0.33	1.91 (1.81-2.01)	6.99E-07	1.92 (1.82-2.02)	6.56E-07
H9 (C-C-G-G)	rs2288491-rs4985534-**rs11645214**-**rs6499323**	0.47	0.33	1.86 (1.76-1.96)	2.08E-06	1.87 (1.77-1.97)	1.76E-06
H10 (C-G-G-T)	rs4985534-**rs11645214**-**rs6499323**-rs7197333	0.47	0.33	1.86 (1.76-1.96)	2.08E-06	1.87 (1.77-1.97)	1.76E-06
H11 (G-G-T-T)	**rs11645214**-**rs6499323**-rs7197333-rs1006985	0.39	0.27	1.74 (1.63-1.86)	4.63E-05	1.76 (1.65-1.88)	3.16E-05
Window size of 5-SNP							
H12 (A-G-C-C-G)	rs2303792-rs8052125-rs2288491-rs4985534-**rs11645214**	0.48	0.33	1.90 (1.80-2.00)	9.62E-07	1.91 (1.81-2.01)	1.15E-06
H13 (G-C-C-G-G)	rs8052125-rs2288491-rs4985534-**rs11645214**-**rs6499323**	0.47	0.33	1.86 (1.76-1.96)	2.08E-06	1.90 (1.80-2.01)	1.48E-06
H14 (C-C-G-G-T)	rs2288491-rs4985534-**rs11645214**-**rs6499323**-rs7197333	0.47	0.33	1.86 (1.76-1.96)	2.08E-06	1.90 (1.80-2.01)	1.48E-06
H15 (C-G-G-T-T)	rs4985534-**rs11645214**-**rs6499323**-rs7197333-rs1006985	0.39	0.27	1.74 (1.63-1.86)	4.72E-05	1.85 (1.74-1.97)	9.85E-06

H-freq: haplotype frequency.

^a^Only crude *p*-value <10^−4^ was presented.

^b^A haplotype with a frequency of >0.05 was included in the analysis.

^c^p-value from logistic regression.

^d^p-value from logistic regression after adjustment of subject's sex, age, BMI, and durations of diabetes.

## Discussion

There is very limited evidence that any of genetic variants contribute to DN in Han Chinese populations. In this study, we expand on the previous investigation by Tsai et al. [15], which described compelling evidence of an association of type 2 diabetes with polymorphisms. We found that p-values of four SNPs were less than 10^−5^ in the Figure 1, and two signals, i.e. p-value <10^−4^, were observed. We identified 11 SNPs in four loci located in 16q22.1 that were associated with DN in a Han Chinese population. As a consequence, we were able to identify novel loci specifically associated with DN. Two SNPs of two loci with p-values in the 10^−5^ to 10^−7^ range were identified for DN. These include SNPs rs11645214 (*SF3B3*) and rs6499323 (*IL34*), with about a 2-fold increased risk of DN. In the haplotype association analysis, 15 haplotypes, including these three SNPs (rs11647932, rs11645214, and rs6499323), carried an approximately 2-fold increased risk of DN, which further confirmed the findings of the single-SNP association tests.

Previous studies using GWAS approach on DN in different racial groups have been conducted [10-14]. These studies showed that different polymorphisms in or near genes may be related to DN susceptibility, for example, intron 18 + 9170 (*ELMO1*) in Japanese with type 2 diabetes [10]; rs2648875 (*PVT1*) and rs2720709 (*PVT1*) in Pima Indians with type 2 diabetes [11]; rs6930576 (*SASH1*), rs7769051 (near *RPS12*), rs2358944 (*MSRB3* ~ *HMGA2*), rs2106294 (*LIMK2*), rs4820043 (*LIMK2*), rs7735506 (*AUH*), and rs5749286 (*SFI1*) in African Americans with type 2 diabetes [12]; rs10868025 (near *FRMD3*), rs1888747 (near *FRMD3*), rs451041 (*CARS*), and rs739401 (*CARS*) in European ancestry with type 1 diabetes [13]; and rs12437854 (*RGMA ~ MCTP2*), rs7583877 (*AFF3*), and rs7588550 (*ERBB4*) in European ancestry with type 1 diabetes [14] (Additional file 1: Table S1). The susceptibility variants for DN varied widely among different population groups, which may be due to ethnic differences and genetic backgrounds. In this study, we found that polymorphisms in the *ST3GAL2*, *COG4*, *SF3B3*, *IL34*, and *BMP7* genes may contribute to DN susceptibility in Han Chinese residing in Taiwan. In previous candidate gene studies, these genes have not been reported for DN in Han Chinese (Additional file 1: Table S2). The proportions of minor alleles of SNPs rs11647932 and rs6499323 among Taiwanese (14% and 34%, respectively) were similar to those of Han Chinese in China (CHB: 14% and 36%), but higher than those of Japanese (JPT: 12% and 28%), Europeans (CEU: 12% and 20%), and Africans (YRI: 0% and 12%) [24].

Many plausible mechanisms have been hypothesized to be involved in the association between DN and identified SNPs. First, the protein encoded by the *ST3GAL2* gene mapped on chromosome 16q22.1 catalyzes the transfer of sialic acid from CMP-sialic acid to galactose-containing substrates [25]. Sialic acid is one of the inflammatory biomarkers of the acute-phase response and is a possible risk factor for cardiovascular disease [26]. Sialic acid was synthesized as a result of cytokines released by inflammatory cells and damaged endothelia due to angiopathy-associated tissue injury [26]. This cytokine response could directly result in vasculopathy in diabetes through atherosclerosis [26]. Moreover, cross-sectional and longitudinal studies have shown that elevated serum sialic acid concentrations are related to diabetic microvascular complications (including DN) in type 2 diabetes [27,28] or type 1 diabetes [29,30]. A strong association between increased serum sialic acid concentrations and macrovascular complications (coronary heart disease; CHD) in type 2 diabetes was also observed [31]. In addition, a 7-year prospective cohort study revealed that a raised serum sialic acid concentration is associated with CHD in type 1 diabetes [32].

Second, the protein encoded by the *COG4* (a component of oligomeric golgi complex 4) gene, including COG4, is critical for the structure and function of the Golgi apparatus and can influence intracellular membrane trafficking, including the addition and processing of carbohydrates (glycosylation) in the rough endoplasmic reticulum and Golgi [33,34]. Mutations in *COG4* may lead to abnormal functioning in the Golgi apparatus, resulting in an excessive amount of glucose circulating in the blood plasma. Diabetes mellitus is characterized by chronic hyperglycaemia. A number of studies have examined the relationship of hyperglycaemia, classified according to mean levels of HbA1c [35-37] or fasting plasma glucose [38,39], with diabetic complications.

Third, the *IL34* (interleukin 34) gene maps on chromosome 16q22.1. Interleukin 34 is a cytokine that promotes the differentiation and viability of monocytes and macrophages through the colony-stimulating factor-1 receptor (CSF1R) [40], and the causative role of macrophages and/or monocytes in DN has been demonstrated by cell depletion studies in animal models [41,42]. Many studies have shown that inflammatory cytokines such as IL-1, IL-6, IL-18, and TNF-α are important in the pathogenesis of diabetic microvascular complications (including DN) [43]. Furthermore, previous studies have revealed that cytokine gene polymorphisms such as *IL-1*, *IL-6*, and *TNF-α* are related to DN [44]. Variants in *IL34* may play a role in the pathogenesis of DN because of the highly correlated cytokine network, although there is no direct evidence for the association between *IL34* gene polymorphisms and DN.

Fourth, the protein encoded by the *BMP7* (bone morphogenetic protein 7) gene mapped on 20q13.3 is a member of the transforming growth factor-beta (TGF-β) superfamily, and is highly expressed in the kidney tubules and glomeruli [45]. Previous studies showed that TGF-β cellular signaling is critical to the induction of glomerular and tubulointerstitial fibrosis in DN [46]. Animal studies demonstrated that the renal BMP7 protected against DN [47], and the BMP7 partially reversed kidney hypertrophy induced by diabetes, urine albumin excretion, restoring GFR, as well as glomerular histology toward normal [48]. McKnight et al. investigated the association of BMP gene variants with DN in White individuals with type 1 diabetes, and they reported that the common variants in *BMP2*, *BMP4* and *BMP7* genes did not strongly influence genetic susceptibility to DN [49]. In our study, we found that two SNPs in *BMP7* gene may be susceptibility variants of DN in Han Chinese patients with type 2 diabetes.

For predicting potential rSNPs, we used the *in silico* prediction tool is-rSNP [23]. The is-rSNP predicted that rs2058289, rs9928626, rs11647932, rs1028555, rs6127999, rs6131015, rs7273764, and rs4815800 are potential rSNPs; that is, they affect the binding affinity of a transcription factor to the DNA (Additional file 1: Table S3). For example, alleles of rs2058289 alter the binding affinity of Sp4_2 (p *= *0.021) and Eomes_1 (p *= *0.036); alleles of rs9928626 alter the binding affinity of LYS14 (p *= *0.018), YKL222C (p *= *0.043), and YDR520C (p *= *0.043); and alleles of rs11647932 alter the binding affinity of LM226 (p *= *0.050). Furthermore, from the online Nephromine database (www.nephromine.org), there were some evidences in the literature that the expression of candidate genes in our study were associated with DN in human population (Additional file 1: Table S4) or mice (Additional file 1: Table S5).

The subjects in this study were a portion of the cohort studied in a published GWAS paper [15]. Although the sample size in this study was limited, the DN cases and the controls were much more homogeneous in terms of gender, age, durations of diabetes, and HbA1c level after excluding subjects with extreme characteristics and matching their age and durations of diabetes. A previous study found that the Han Chinese residing in Taiwan were relatively homogenous in genetic background, spread among the three major ethnic groups of Minnan, Hakka, and Mainland Chinese [50], which is consistent with the results of our population stratification analysis. Therefore, the impact of population stratification on our genetic association study is small.

## Conclusions

We identified 3 novel SNPs (rs11647932, rs11645214, and rs6499323) and 11 haplotypes (4, 3, and 4 in window size of 3-SNP, 4-SNP, and 5-SNP, respectively), located at the 16q22.1 region, that are susceptibility variants of DN in a Han Chinese population in Taiwan. The findings reveal that the susceptibility variants located at the 16q22.1 region may involve in the biological pathways of DN in Han Chinese patients with type 2 diabetes. Our results can provide new insights into the etiology of DN.

## Additional file

Additional file 1: Figure S1. Regional plot of these 11 SNPs located on chromosome 16q22.1. The –log_10 _(p-value) (left y-axis) was from the trend test using our data. The estimated *r* ^*2*^ and recombination rate (right y-axis) based on the HapMap Phase II JPT + CHB populations were plotted to reflect the LD structure. The gene information was from the UCSC (Build hg18). The regional plot was plotted from the LocusZoom, a web-based plotting tool (csg.sph.umich.edu/locuszoom). **Table S1.** Summary of identified polymorphisms through GWAS. **Table S2.** Summary of variants associated with nephropathy in Han Chinese patients with type 2 diabetes through candidate gene approach. **Table S3.** is-rSNP prediction results^a^. **Table S4.** Gene expression from human diabetic nephropathy datasets in the Nephromine database. **Table S5.** Gene expression from Hodgin Diabetes Mouse dataset^a^ in the Nephromine database [51-63].

## Abbreviations

DN: Diabetic nephropathy
ESRD: End-stage renal disease
CKD: Chronic kidney disease
eGFR: Estimated glomerular filtration rate
HDL-C: Hhigh-density lipoprotein cholesteroll
LDL-C: Low-density lipoprotein cholesterol
BUN: Blood urea nitrogen
MDS: Multidimensional scaling
LD: Linkage disequilibrium
