# Supplementary material for: Identified single-nucleotide polymorphisms and haplotypes at 16q22.1 increase diabetic nephropathy risk in Han Chinese population
Source: BMC Genet. 2014 Oct 31;15:113. doi: 10.1186/s12863-014-0113-8 (PMC4222374; doi:10.1186/s12863-014-0113-8)
Supplement: Additional file 1: Figure S1. — Regional plot of these 11 SNPs located on chromosome 16q22.1. The –log10 (p-value) (left y-axis) was from the trend test using our data. The estimated r 2 and recombination rate (right y-axis) based on the HapMap Phase II JPT + CHB populations were plotted to reflect the LD structure. The gene information was from the UCSC (Build hg18). The regional plot was plotted from the LocusZoom, a web-based plotting tool (csg.sph.umich.edu/locuszoom). Table S1. Summary of identified polymorphisms through GWAS. Table S2. Summary of variants associated with nephropathy in Han Chinese patients with type 2 diabetes through candidate gene approach. Table S3. is-rSNP prediction resultsa. Table S4. Gene expression from human diabetic nephropathy datasets in the Nephromine database. Table S5. Gene expression from Hodgin Diabetes Mouse dataseta in the Nephromine database [51-63]. [file 12863_2014_113_MOESM1_ESM.docx]

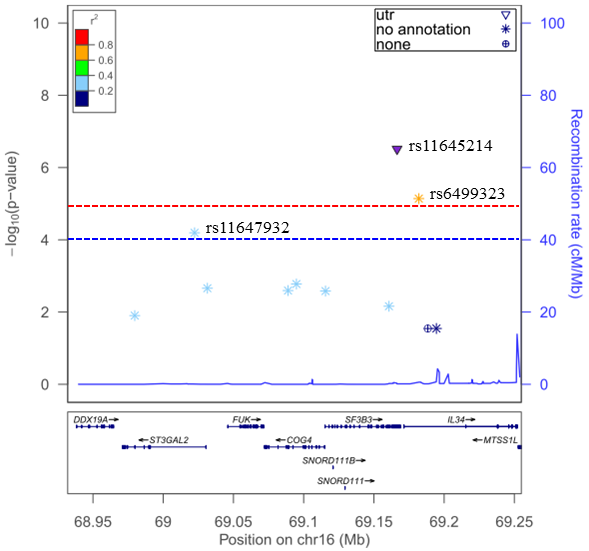


**Supplemental Figure 1. Regional plot of these 11 SNPs located on chromosome 16q22.1.** The –log_10_(p-value) (left y-axis) was from the trend test using our data. The estimated *r^2^* and recombination rate (right y-axis) based on the HapMap Phase II JPT+CHB populations were plotted to reflect the LD structure. The gene information was from the UCSC (Build hg18). The regional plot was plotted from the LocusZoom, a web-based plotting tool (csg.sph.umich.edu/locuszoom).

Supplemental Table 1. Summary of identified polymorphisms through GWAS

| SNP | Gene | Location | Phenotype | Population | OR (95% CI) | P-value | Reference |
| --- | --- | --- | --- | --- | --- | --- | --- |
| **Type 2 diabetes** | |  |  |  |  |  |  |
| intron 18+9170 | ELMO1 | 7p14.1 | Proteinuria/ESRD | Japanese | 2.67 (1.71–4.16) | 8.00E-06 | Shimazaki A et al., 2005 [10]. |
| rs2648875 | PVT1 | 8q24 | ESRD | Pima Indians | 2.97 (1.90–4.65) | 1.80E-06 | Hanson RL et al., 2007 [11]. |
| rs2720709 | PVT1 | 8q24 | ESRD | Pima Indians | 2.57 (1.66–3.96) | 2.10E-05 |  |
| rs6930576 | SASH1 | 6q24.3 | ESRD | African Americans | 1.31 (1.18–1.45) | 7.04E-07 | McDonough CW et al., 2011 [12]. |
| rs7769051 | RPS12 | 6q23.2 | ESRD | African Americans | 1.28 (1.16–1.42) | 2.20E-06 |  |
| rs2358944 | MSRB3~HMGA2 | 12q14.3 | ESRD | African Americans | 0.75 (0.67–0.85) | 3.54E-06 |  |
| rs2106294 | LIMK2 | 22q12.2 | ESRD | African Americans | 0.57 (0.45–0.72) | 4.11E-06 |  |
| rs4820043 | LIMK2 | 22q12.2 | ESRD | African Americans | 0.57 (0.45–0.73) | 5.07E-06 |  |
| rs773506 | AUH | 9q22.3 | ESRD | African Americans | 0.76 (0.67–0.85) | 6.45E-06 |  |
| rs5749286 | SFI1 | 22q12.2 | ESRD | African Americans | 0.60 (0.47–0.75) | 9.79E-06 |  |
| rs11645214 | SF3B3 | 16q22.1 | Proteinuria/CKD | Han Chinese | 1.92 (1.49–2.48) | 3.07E-07 | Our study |
| rs10963767 | ADAMTSL1 | 9p21.3 | Proteinuria/CKD | Han Chinese | 1.83 (1.41–2.36) | 3.46E-06 |  |
| rs2058289 | TMEM132B~TMEM132C | 12q24.3 | Proteinuria/CKD | Han Chinese | 2.08 (1.51–2.88) | 6.11E-06 |  |
| rs6499323 | IL34 | 16q22.1 | Proteinuria/CKD | Han Chinese | 1.77 (1.38–2.28) | 7.24E-06 |  |
| rs11647932 | ST3GAL2 | 16q22.1 | Proteinuria/CKD | Han Chinese | 1.84 (1.36–2.50) | 6.39E-05 |  |
| **Type 1 diabetes** | |  |  |  |  |  |  |
| rs10868025 | FRMD3 | 9q21.3 | Macroalbuminuria/ESRD | European ancestry | 1.45 (1.25–1.67) | 5.00E-07 | Pezzolesi MG et al., 2009 [13]. |
| rs1888747 | FRMD3 | 9q21.3 | Macroalbuminuria/ESRD | European ancestry | 1.45 (1.25–1.67) | 6.30E-07 |  |
| rs451041 | CARS | 11p15.5 | Macroalbuminuria/ESRD | European ancestry | 1.36 (1.19–1.56) | 3.10E-06 |  |
| rs739401 | CARS | 11p15.5 | Macroalbuminuria/ESRD | European ancestry | 1.36 (1.19–1.56) | 6.40E-06 |  |
| rs12437854 | RGMA~MCTP2 | 15q26 | Macroalbuminuria/ESRD | European ancestry | 1.8 (1.48–2.17) | 2.00E-09 | Sandholm N et al., 2012 [14]. |
| rs7583877 | AFF3 | 2q11.2-q12 | Macroalbuminuria/ESRD | European ancestry | 1.29 (1.18–1.40) | 1.20E-08 |  |
| rs7588550 | ERBB4 | 2q33.3-q34 | Macroalbuminuria/ESRD | European ancestry | 0.66 ( 0.56–0.77) | 2.10E-07 |  |

Supplemental Table 2. Summary of variants associated with nephropathy in Han Chinese patients with type 2 diabetes through candidate gene approach

| Variant | Gene | Location | OR (95% CI) | P-value | Reference |
| --- | --- | --- | --- | --- | --- |
| XbaI | GLUT1 | 1p34.2 | 1.92 (1.04–3.51) | p < 0.05 | Liu ZH et al., 1999 [51]. |
| I/D | ACE | 17q23.3 | － | p < 0.05 | Hsieh MC et al., 2000 [52]. |
| A2350G | ACE | 17q23.3 | 1.47 (1.21–1.79) | p < 0.0001 | Huo P et al., 2014 [53]. |
| Val16Ala | SOD2 | 6q25.3 | 0.42 (0.18–0.95) | 0.037 | Liu L et al., 2009 [54]. |
| rs2268388 | ACACB | 12q24.11 | 1.07 (0.78–1.48) | 0.640 | Maeda S et al., 2010 [55]. |
| rs1801282 | PPARG | 3p25 | 2.30 (1.18–4.45) | 0.014 | Liu L et al., 2010 [56]. |
| rs2268388 | ACACB | 12q24.11 | 2.39 (1.20 – 4.75) | 0.129 | Tang SC et al., 2010 [57]. |
| BsmI | VDR | 12q13.11 | － | 0.007 | Zhang H et al., 2012 [58]. |
| rs1143770 | let-7a-2 | 11q24.1 | － | p < 0.01 | Zhou J et al., 2013 [59]. |
| rs741301 | ELMO1 | 7p14.1 | OR: 1.89 | 0.004 | Wu HY et al., 2013 [60]. |
| rs10951509 | ELMO1 | 7p14.1 | OR: 1.76 | 0.020 | Wu HY et al., 2013 [60]. |
| G894T | eNOS | 7q36 | 0.47 (0.34–0.66) | p < 0.0001 | Huo P et al., 2014 [53]. |

Supplemental Table 3. is-rSNP prediction results^a^

| Location | SNP | Transcription factor | Adjusted p-value^b^ |
| --- | --- | --- | --- |
| **SNPs with p-value < 10^-5^** | | | |
| 12q24.3 | rs2058289 | Sp4_2 | **0.021** |
| 12q24.3 | rs2058289 | Eomes_1 | **0.036** |
| 12q24.3 | rs2058289 | T | 0.058 |
| 12q24.3 | rs2058289 | Obox1 | 0.083 |
| 16q22.1 | rs11645214 | Rxra_2 | 0.056 |
| 16q22.1 | rs11645214 | GATAAGR | 0.083 |
| **Signal on chromosome 16 (p-value < 10^-4^)^c^** | | | |
| 16q21 | rs9928626 | LYS14 | **0.018** |
| 16q21 | rs9928626 | YKL222C | **0.043** |
| 16q21 | rs9928626 | YDR520C | **0.043** |
| 16q21 | rs9928626 | LM80 | 0.085 |
| 16q22.1 | rs11647932 | LM226 | **0.050** |
| 16q21 | rs876142 | LM192 | 0.074 |
| 16q22.1 | rs11645214 | Rxra_2 | 0.085 |
| **Signal on chromosome 20 (p-value < 10^-4^)^d^** | | | |
| 20p12.3 | rs1028555 | YNTTTNNNANGCARM | **0.003** |
| 20p12.3 | rs1028555 | Pou5f1 | **0.025** |
| 20q13.3 | rs6127999 | TCCCRNNRTGC | **0.028** |
| 20q13.1 | rs6131015 | LM9 | **0.030** |
| 20q13.3 | rs7273764 | PPARG::RXRA | **0.030** |
| 20p12.3 | rs4815800 | Irx5 | **0.037** |
| 20p12.3 | rs4815800 | Irx2 | 0.057 |
| 20q13.3 | rs1885580 | LM15 | 0.059 |

a: From the Manhattan plot in Figure 1, there were four SNPs (rs10963767, rs2058289, rs11645214, and rs6499323) with p-value < 10^-5^ (above the red line), and two signals with p-value < 10^-4^ on chromosomes 16 and 20 were observed. We used the is-rSNP tool to predict potential regulatory SNPs by separately entering three blocks of SNPs in this table, and we only reported adjusted p-values < 0.10 for each SNP in each part.

b: Adjusted p-values were calculated by using Benjamini-Hochberg method. Adjusted p-values < 0.05 were highlighted in bold.

c: Five SNPs were analyzed, including rs876142, rs9928626, rs11647932, rs11645214, and rs6499323.

d: Fifteen SNPs were analyzed, including rs1028555, rs4815800, rs6065925, rs6131015, rs4812997, rs6074024, rs6127983, rs182784, rs1885580, rs7273764, rs6127999, rs6014975, rs4811839, rs6025517, and rs2426712.

Supplemental Table 4. Gene expression from human diabetic nephropathy datasets in the Nephromine database

| Gene | Tissue | Diabetic Nephropathy | Healthy Living Donor | Cadaveric Donor Control | P-value | Fold Change | Dataset |
| --- | --- | --- | --- | --- | --- | --- | --- |
| **Under-expression** | |  |  |  |  |  |  |
| ST3GAL2 | － | 11 | 3 | 4 | 2.00E-03 | -1.13 | Schmid Diabetes [61] |
| ST3GAL2 | Tubulointerstitium | 17 | 41 | － | 9.00E-03 | -1.16 | Ju Podocyte* |
| BMP7 | Glomeruli | 9 | 13 | － | 1.35E-07 | -3.65 | Woroniecka Diabetes [62] |
| BMP7 | Tubulointerstitium | 17 | 41 | － | 8.24E-06 | -1.69 | Ju Podocyte* |
| BMP7 | Tubulointerstitium | 10 | 12 | － | 3.32E-05 | -1.53 | Woroniecka Diabetes [62] |
| BMP7 | Glomeruli | 12 | 41 | － | 4.23E-04 | -2.03 | Ju Podocyte* |
| BMP7 | － | 11 | 3 | 4 | 7.00E-03 | -1.14 | Schmid Diabetes [61] |
| **Over-expression** | |  |  |  |  |  |  |
| COG4 | Tubulointerstitium | 17 | 41 | － | 1.50E-02 | 1.09 | Ju Podocyte* |
| SF3B3 | Glomeruli | 12 | 41 | － | 1.70E-02 | 1.10 | Ju Podocyte* |

*: Not yet published, 2013/05/21.

Schmid Diabetes and Ju Podocyte datasets: gene expression levels (mRNA) of samples were analyzed on Affymetrix Human Genome U133A Array.

Woroniecka Diabetes dataset: gene expression levels (mRNA) of samples were analyzed on Affymetrix Human Genome U133A 2.0 Array.

Supplemental Table 5. Gene expression from Hodgin Diabetes Mouse dataset^a^ in the Nephromine database

| Gene | Model | Diabetic Nephropathy Mouse Model | Non-Diabetic Mouse Kidney | P-value | Fold Change |
| --- | --- | --- | --- | --- | --- |
| **Under-expression** | |  |  |  |  |
| COG4 | eNOS-deficient C57BLKS db/db Model | 7 | 5 | 6.00E-03 | -1.15 |
| COG4 | db/db C57BLKS Model | 5 | 5 | 1.10E-02 | -1.16 |
| IL34 | DBA/2 Model | 9 | 8 | 1.70E-02 | -1.28 |

a: Hodgin Diabetes Mouse dataset [63]: mRNA levels; platform not pre-defined in Oncomine.

**References**

51. Liu ZH, Guan TJ, Chen ZH, Li LS: Glucose transporter (GLUT1) allele (XbaI-) associated with nephropathy in non-insulin-dependent diabetes mellitus. Kidney Int 1999, 55:1843-1848.

52. Hsieh MC, Lin SR, Hsieh TJ, Hsu CH, Chen HC, Shin SJ, Tsai JH: Increased frequency of angiotensin-converting enzyme DD genotype in patients with type 2 diabetes in Taiwan. Nephrol Dial Transplant 2000, 15:1008-1013.

53. Huo P, Zhang D, Guan X, Mei Y, Zheng H, Feng X: Association between genetic polymorphisms of ACE & eNOS and diabetic nephropathy. Molecular biology reports 2014.

54. Liu L, Zheng T, Wang N, Wang F, Li M, Jiang J, Zhao R, Li L, Zhao W, Zhu Q, Jia W: The manganese superoxide dismutase Val16Ala polymorphism is associated with decreased risk of diabetic nephropathy in Chinese patients with type 2 diabetes. Mol Cell Biochem 2009, 322:87-91.

55. Maeda S, Kobayashi MA, Araki S, Babazono T, Freedman BI, Bostrom MA, Cooke JN, Toyoda M, Umezono T, Tarnow L, et al: A single nucleotide polymorphism within the acetyl-coenzyme A carboxylase beta gene is associated with proteinuria in patients with type 2 diabetes. PLoS Genet 2010, 6:e1000842.

56. Liu L, Zheng T, Wang F, Wang N, Song Y, Li M, Li L, Jiang J, Zhao W: Pro12Ala polymorphism in the PPARG gene contributes to the development of diabetic nephropathy in Chinese type 2 diabetic patients. Diabetes Care 2010, 33:144-149.

57. Tang SC, Leung VT, Chan LY, Wong SS, Chu DW, Leung JC, Ho YW, Lai KN, Ma L, Elbein SC, et al: The acetyl-coenzyme A carboxylase beta (ACACB) gene is associated with nephropathy in Chinese patients with type 2 diabetes. Nephrol Dial Transplant 2010, 25:3931-3934.

58. Zhang H, Wang J, Yi B, Zhao Y, Liu Y, Zhang K, Cai X, Sun J, Huang L, Liao Q: BsmI polymorphisms in vitamin D receptor gene are associated with diabetic nephropathy in type 2 diabetes in the Han Chinese population. Gene 2012, 495:183-188.

59. Zhou J, Peng R, Li T, Luo X, Peng H, Zha H, Yin P, Wen L, Zhang Z: A potentially functional polymorphism in the regulatory region of let-7a-2 is associated with an increased risk for diabetic nephropathy. Gene 2013, 527:456-461.

60. Wu HY, Wang Y, Chen M, Zhang X, Wang D, Pan Y, Li L, Liu D, Dai XM: Association of ELMO1 gene polymorphisms with diabetic nephropathy in Chinese population. Journal of endocrinological investigation 2013, 36:298-302.

61. Schmid H, Boucherot A, Yasuda Y, Henger A, Brunner B, Eichinger F, Nitsche A, Kiss E, Bleich M, Grone HJ, et al: Modular activation of nuclear factor-kappaB transcriptional programs in human diabetic nephropathy. Diabetes 2006, 55:2993-3003.

62. Woroniecka KI, Park AS, Mohtat D, Thomas DB, Pullman JM, Susztak K: Transcriptome analysis of human diabetic kidney disease. Diabetes 2011, 60:2354-2369.

63. Hodgin JB, Nair V, Zhang H, Randolph A, Harris RC, Nelson RG, Weil EJ, Cavalcoli JD, Patel JM, Brosius FC, 3rd, Kretzler M: Identification of cross-species shared transcriptional networks of diabetic nephropathy in human and mouse glomeruli. Diabetes 2013, 62:299-308.
